# Optic Nerve Diffusion Tensor Imaging after Acute Optic Neuritis Predicts Axonal and Visual Outcomes

**DOI:** 10.1371/journal.pone.0083825

**Published:** 2013-12-26

**Authors:** Anneke van der Walt, Scott C. Kolbe, Yejun E. Wang, Alexander Klistorner, Neil Shuey, Gelareh Ahmadi, Mark Paine, Mark Marriott, Peter Mitchell, Gary F. Egan, Helmut Butzkueven, Trevor J. Kilpatrick

**Affiliations:** 1 Department of Anatomy and Neuroscience, University of Melbourne, Melbourne, Victoria, Australia; 2 Department of Neurology, Royal Melbourne Hospital, Melbourne, Victoria, Australia; 3 Department of Neuro-ophthalmology, Royal Victorian Eye and Ear Hospital, Melbourne, Victoria, Australia; 4 Save Sight institute, Sydney University, Sydney, New South Wales, Australia; 5 Department of Radiology, Royal Melbourne Hospital, Melbourne, Victoria, Australia; 6 Monash University, Melbourne, Victoria, Australia; 7 Melbourne Brain Centre at Royal Melbourne Hospital, University of Melbourne, Melbourne, Victoria, Australia; Institute Biomedical Research August Pi Sunyer (IDIBAPS) - Hospital Clinic of Barcelona, Spain

## Abstract

**Background:**

Early markers of axonal and clinical outcomes are required for early phase testing of putative neuroprotective therapies for multiple sclerosis (MS).

**Objectives:**

To assess whether early measurement of diffusion tensor imaging (DTI) parameters (axial and radial diffusivity) within the optic nerve during and after acute demyelinating optic neuritis (ON) could predict axonal (retinal nerve fibre layer thinning and multi-focal visual evoked potential amplitude reduction) or clinical (visual acuity and visual field loss) outcomes at 6 or 12 months.

**Methods:**

Thirty-seven patients presenting with acute, unilateral ON were studied at baseline, one, three, six and 12 months using optic nerve DTI, clinical and paraclinical markers of axonal injury and clinical visual dysfunction.

**Results:**

Affected nerve axial diffusivity (AD) was reduced at baseline, 1 and 3 months. Reduced 1-month AD correlated with retinal nerve fibre layer (RNFL) thinning at 6 (R=0.38, p=0.04) and 12 months (R=0.437, p=0.008) and VEP amplitude loss at 6 (R=0.414, p=0.019) and 12 months (R=0.484, p=0.003). AD reduction at three months correlated with high contrast visual acuity at 6 (ρ = -0.519, p = 0.001) and 12 months (ρ = -0.414, p=0.011). The time-course for AD reduction for each patient was modelled using a quadratic regression. AD normalised after a median of 18 weeks and longer normalisation times were associated with more pronounced RNFL thinning and mfVEP amplitude loss at 12 months. Affected nerve radial diffusivity (RD) was unchanged until three months, after which time it remained elevated.

**Conclusions:**

These results demonstrate that AD reduces during acute ON. One month AD reduction correlates with the extent of axonal loss and persistent AD reduction at 3 months predicts poorer visual outcomes. This suggests that acute ON therapies that normalise optic nerve AD by 3 months could also promote axon survival and improve visual outcomes.

## Introduction

Multiple sclerosis (MS) is the most common neurological disorder affecting young Caucasians, with a higher prevalence in women. The disease is characterised pathologically by immune attack against myelin and axons within the central nervous system (CNS). Regions of neuroinflammation (called lesions) can be visualised using T2-weighted magnetic resonance imaging (MRI) [1]. A single lesion is rarely associated with a clinical relapse unless present in pathways such as the spinal cord or optic nerves that are critical for transmitting sensory or motor signals to and from the brain. Inflammation of the optic nerve, called optic neuritis (ON), results in acute vision loss that prompts patients to present early to medical attention. Furthermore, visual acuity loss is readily measurable and reliable resulting in early diagnosis. . As such, the optic nerve has become a structure of significant interest for studies aiming to identify early imaging markers that predict clinical and neurodegenerative outcomes [2]. Such imaging markers could find use in phase II clinical trials of novel neuroprotective therapies [3].

Several markers can inform the extent of optic nerve injury after ON. Optical coherence tomography (OCT) is a technique that images the retinal nerve fibre layer (RNFL). The RNFL is unique in that it comprises unmyelinated axons from retinal ganglion cells and thinning of the RNFL is, therefore, considered to be a relatively pure marker for optic nerve axonal atrophy and degeneration [4]. Visual evoked potentials (VEP) can reveal both optic nerve demyelination (prolongation of VEP latency) and axonal loss (reduction of VEP amplitude) after acute ON [5]. A more recently developed technique, that records potentials associated with stimulation of wider regions of the visual field, multifocal VEP (mfVEP), can reveal sectorial demyelination or degeneration after ON [6-8] as well as in MS subgroups [9,10]. While both OCT and mfVEP are excellent methods for assessing post acute optic nerve injury, both techniques are acutely affected by inflammation and therefore cannot be used to assess the extent of acute axonal injury. 

Diffusion tensor imaging (DTI), an MRI technique sensitive to water molecular diffusion in white matter, has recently been shown to respond at the earliest stages of ON [11,12]. DTI enables the quantification of axial and radial diffusion coefficients. In animal models, histopathologically verified axonal injury is associated with reduced axial diffusion (AD) within hours of acute injury followed by subsequent normalisation [13,14]. Recent translational work has suggested that acutely reduced AD could be predictive of subsequent optic nerve axonal degeneration and visual outcomes after ON [11]. However, assessing the veracity of these findings requires further investigation in an independent cohort.

This study therefore aimed to assess longitudinal changes in optic nerve AD during and after acute ON, and to evaluate correlations between these changes and axonal and clinical outcomes at 6 and 12 months after the acute event. We hypothesised that AD would be acutely attenuated. We also predicted that the degree of acute attenuation of optic nerve AD would correlate with the degree of subsequent axonal loss measured using OCT and mfVEP, and the degree of subsequent vision loss measured using high and low contrast visual acuity charts. To test these hypotheses, we recruited patients with acute ON at high risk of developing MS and tested them at the earliest stages of the disease with subsequent follow-up at one, three, six and 12 months.

## Materials and Methods

### Subjects and study protocol

Forty adults (18 to 60 years) presenting within two weeks of symptom onset with a first episode of unilateral ON were recruited consecutively between 2008 and 2010 from a tertiary ophthalmology hospital. All patients had at least two T2-hyperintense lesions on a baseline MRI scan indicating a high risk of subsequent development of MS (87% over the ensuing ten years) [15]. We excluded patients with other neurological or ophthalmological diseases or further episodes of ON during the study period. Patients with early relapsing remitting MS (onset within two years) according to MRI-based criteria [16] were included. Patients were studied at baseline (within 48 hours of presentation and in all cases prior to steroid administration) and at one, three, six and 12 months (range of two weeks) after onset. Optic neuritis was diagnosed by standard clinical criteria [17]. Two patients elected to withdraw from the study at one month and one patient was excluded due to recurrent ON. Missed appointments included one patient at one month and two patients at six months. Diagnosis in atypical presentations was corroborated by optic nerve gadolinium enhancement (indicating acute inflammation) or abnormal mfVEP. Ten healthy volunteers were recruited and tested twice, four weeks apart, to assess inter-scan variability in optic nerve DTI. 

This study was conducted in accordance with the Declaration of Helsinki and was approved by the Human Research Ethics Committees of the Royal Victorian Eye and Ear Hospital and the Royal Melbourne Hospital. All study participants provided voluntary, written consent.

### Clinical assessments

Best corrected visual acuity (VA) was measured using Sloan high contrast letter acuity charts (HCLA) (100%) and low contrast letter acuity charts (LCLA) (2.5%) viewed at 2m in a room well lit by white fluorescent lighting [18,19]. For HCLA and LCLA, the numbers of letters correctly identified (maximum 60/chart) were recorded for each eye. Colour vision was recorded as correct Ishihara plates out of 38 [20]. A detailed ophthalmological examination was performed at baseline. Neurological assessments included Expanded Disability Status Scale (EDSS) [21] scores at baseline, six and 12 months performed by a neurologist experienced in MS clinical management. 

### Optical Coherence Tomography (OCT)

OCT was performed using an OCT-3 scanner (Stratus™, software version 3.0, Carl ZeissMeditec Inc.) using the Fast RNFL protocol consisting of three circular 3.4 mm diameter scans centred on the optic disc. Signal strength of seven or more was deemed acceptable. 

### Multi-focal Visual Evoked Potentials (mfVEP)

The mfVEP was performed using the Accumap™ (ObjectiVision, software: Opera, Sydney, Australia) with a previously described testing procedure [7] that entailed recordings from 58 sectors of the visual field using four electrodes placed over the inion on the rear of the skull. Eyes were individually stimulated for 10 to 12 runs until a sufficient signal to noise ratio (SNR) was reached. Mean amplitude was calculated per visual field sector and then per eye as a whole. 

### Magnetic Resonance Imaging and Image Analysis

MRI was performed using a Siemens 3T system with a 32-channel transmit-receive head coil. Optic nerve DTI was acquired using a previously described protocol [22]. Each optic nerve was scanned individually using six-direction DTI acquired with the slice plain orientated orthogonal to the intra-orbital section of each nerve. Imaging parameters were as follows: TR/TE/TI = 3900/79/2100ms; NEX = 22; *b*-value = 600smm^-2^; matrix = 168x168; FOV = 220x220mm^2^; slice thickness = 3.5mm; acquisition time = 5m 10s for each nerve). Subjects were instructed to perform a simple eye fixation task during DTI acquisition to minimise movement of the optic nerve during scanning. 

DTI parameters (AD and RD) were calculated from raw, eddy-current corrected diffusion weighted scans using custom MATLAB® scripts. Regions of interest (ROIs) within the optic nerves were manually delineated on raw diffusion-weighted images as previously described [22]. ROIs were projected onto the calculated DTI maps and the mean AD and RD was calculated for each coronal oblique slice. Data from slices three to seven (intra-orbital optic nerve) were selected for analysis as they have been shown to be most sensitive and can be reliably identified in all subjects [22]. Two independent observers rated 20 randomly selected patient nerves twice, three months apart. 

### Statistical Analyses

The reliability of optic nerve DTI ROI placement was assessed within and between raters using the Bland and Altman limits of agreement method [23]. Within and between subject coefficients of variation (CV) for AD and RD were calculated from control data. Mean and 95% confidence intervals (CI) for healthy optic nerve AD and RD used in subsequent group comparisons were obtained from 5000 bootstrap samples of means calculated from a random nerve (left or right) from a random scan (first or second) from each subject. 

Group distributions for continuous measures were tested for normality using Kolmogorov–Smirnov (K–S) tests. Unaffected nerve AD and RD were compared to control distributions at each time point using unpaired Student’s t-tests. Longitudinal changes in unaffected nerve DTI measures were tested for using one-way repeated-measures Analysis of Variance (ANOVA). Two-way mixed-effects ANOVA was used to test for a significant interaction between time-point and optic nerve clinical status with a subject-level random factor. Significant time-clinical status interactions were further investigated using post-hoc paired t-tests between affected and unaffected nerve DTI parameters at each time point.

Normalised asymmetry coefficients [(affected - unaffected) / unaffected] were calculated for all measures for use in correlation analyses to minimize inter-subject variability not related to disease [7]. All correlation analyses comparing optic nerve DTI parameters and mfVEP or OCT parameters were performed using Pearson parametric correlation procedures. Correlation analyses comparing optic nerve DTI parameters and clinical visual function parameters were performed using Spearman rank correlation procedures. The following correlations were performed: a) early DTI parameters were compared to concurrent mfVEP and clinical visual function parameters, and b) early DTI parameters were compared to six and 12 month OCT, mfVEP and clinical visual function outcomes. To account for multiple correlations, a more stringent level of significance, p<0.01, was applied. 

To further investigate persistent reduction in AD as a predictor of axonal degeneration and visual outcome, individual patient AD asymmetry time-courses were modelled using quadratic functions fit using MATLAB® curve fitting toolbox and linear least-squares fitting procedures. Quadratic fits were chosen because the average time course for AD asymmetry for patients was best fit by a quadratic function (see Results). For each patient, we calculated the time to normalisation (asymmetry = 0) in weeks based on the quadratic fits. For cases where there was no reduction in AD the normalisation time was set to 0 weeks. In two cases, AD remained reduced for the entire 12 months so the normalisation time was set to 52 weeks. Time to normalisation was then correlated with axonal and visual outcomes at 12 months using Spearman’s rank correlations. Rank correlations were chosen over parametric correlations to allow for arbitrarily set minimum (0 weeks) and maximum (52 weeks) values.

Statistical analyses were performed using IBM SPSS Statistics 20. 

## Results

### Patient Demographics (Table 1)

**Table 1:** Baseline demographics of acute optic neuritis patients who completed 12 months of follow- up.

**Characteristic**	n=37
Age (years) (mean, SD)	35 (9)
Female (N,%)	26 (70)
Symptom duration prior to baseline testing (median days, range)	5 (1-14)
Methylprednisolone treatment (N,%)	28 (76)
**Diagnosis at presentation**	
Clinically isolated syndrome (N,%)	31 (84)
Early RRMS* (N,%)	6 (16)
**Clinical Outcome**	
Conversion to Clinically Definite MS by 12 months (N,%)	13 (42)
EDSS at 12 months (median, IQR)	0 (0 to 0.75).
**Baseline visual acuity**	
LogMAR Visual acuity derived from Sloan	0.61 (0.36, 0.25 to 1.0)
100% contrast letter acuity chart (mean, SD, IQR range)	
Low contrast letter acuity** (mean number	3.6 (9.0, 0 to 0)
of letters correct out of 60, SD, IQR)	
Colour vision (mean correct number Ishihara plates out of 38, SD, IQR)	13.1 (14.2, 0 to 29)
**Visual acuity at 12 months**	
LogMAR Visual acuity derived from Sloan	-0.1 (0.19, -0.2 to 0.0)
100% contrast letter acuity chart (mean, SD, IQR)	
Low contrast letter acuity** (mean number of letters	27 (14.6, 20 to 36)
correct out of 60, SD, IQR)	
Colour vision (correct number Ishihara plates out of 38, SD, IQR)	35.7 (7.4, 37 to 38)

^*^ Patients with RRMS with disease duration less than 2 years

^**^ Sloan charts, 2.5% contrast

RRMS = relapsing remitting MSEDSS = expanded disability status scale scoreSD = standard deviation IQR = interquartile range

Baseline and 12 month visual characteristics are shown in Table 1. Thirteen CIS patients (42%) had a second non-ON relapse during the study. No patient was on disease modifying treatment for MS at baseline but 29.7% (n=11) were treated by 6 months and 37.8% (n=14) by 12 months. 

### Reproducibility analyses

Intra-rater reproducibility in ROI placement was high, with the mean difference in AD (normalised to the group mean) of 0.96% (SD 2.8, 95% limits of agreement -4.6 to 6.5), and mean difference in RD of 0.51% (4.2, -7.62 to 8.64). Inter-rater reproducibility was similarly high for both AD (1%, 4.2, -7.20 to 9.23) and RD (0.6%, 7.3, -13.7 to 14.9). Intra-subject CV for control optic nerves was 2.4% for AD and 3% for RD. All optic nerve DTI parameters were normally distributed based on within-group K-S tests.

### Comparison between control nerves and patient unaffected nerves (Table 2)

**Table 2:** Summary statistics for optic nerve DTI (mean* with 95% CI).

	Axial Diffusivity			Radial diffusivity		
	Unaffected eye**	Affected eyeΨ	Inter-ocular difference	Unaffected eye**	Affected eyeΨ	Inter-ocular difference
	1.80	1.54	-0.27	0.93	0.88	-0.05
Baseline	(1.72 to 1.85)	(1.44 to 1.61)	(-0.35 to -0.19)	(-.84 to 0.97)	(0.82 to 0.93)	(0.09 to 0.02)
	p = 0.90	p < 0.001		p = 0.73	p = 0.10	
	1.81	1.61	-0.20	0.93	0.91	-0.02
1 month	(1.75 to 1.86)	(1.54 to 1.67)	(-0.27 to -0.13)	(0.87 to 0.97)	(0.84 to 0.96)	(-0.07 to 0.02)
	p = 0.91	p < 0.001		p = 0.82	p = 0.25	
	1.84	1.74	-0.10	0.94	0.99	0.05
3 months	(1.75 to 1.88)	(1.63 to 1.80)	(-0.16 to – 0.04)	(0.86 to 0.97)	(0.92 to 1.05)	(0.03 to 0.11)
	p = 0.59	p = 0.003		p = 0.96	p = 0.023	
	1.79	1.77	-0.02	0.92	1.0	0.08
6 months	(1.71 to 1.82)	(1.71 to 1.81)	(-0.06 to 0.05)	(0.85 to 0.96)	(0.94 to 1.05)	(0.04 to 0.14)
	p = 0.61	p = 0.74		p = 0.62	p = 0.003	
	1.76	1.82	0.06	0.93	1.07	0.16
12 months	(1.68 to 1.80)	(1.75 to 1.86)	(0.02 to 0.12)	(0.84 to 0.95)	(1.01 to 1.11)	(0.11 to 0.22)
	p = 0.26	p = 0.013		p = 0.44	p < 0.001	

* Measurement units are x10**^*-*^** ^3^ mm^2^s^-1^.

^**^ Significance values from unpaired Student’s t-tests between unaffected nerve and control nerves

Ψ Significance values paired Student’s t-tests between affected and unaffected nerves.

Bootstrapped mean and 95% CI for control AD and RD were 1.78 (1.69 to 1.87) and 0.92 (0.83 to 1.01) respectively. AD and RD did not differ between unaffected nerves and controls at any time point. Unaffected nerve AD (F_4,177_ = 0.969, *p* = 0.426) and RD (F_4,177_ = 0.109, *p* = 0.979) were both stable over 12 months.

### Longitudinal changes in optic nerve DTI over 12 months (Figure 1 and Table 2)

**Figure 1:**
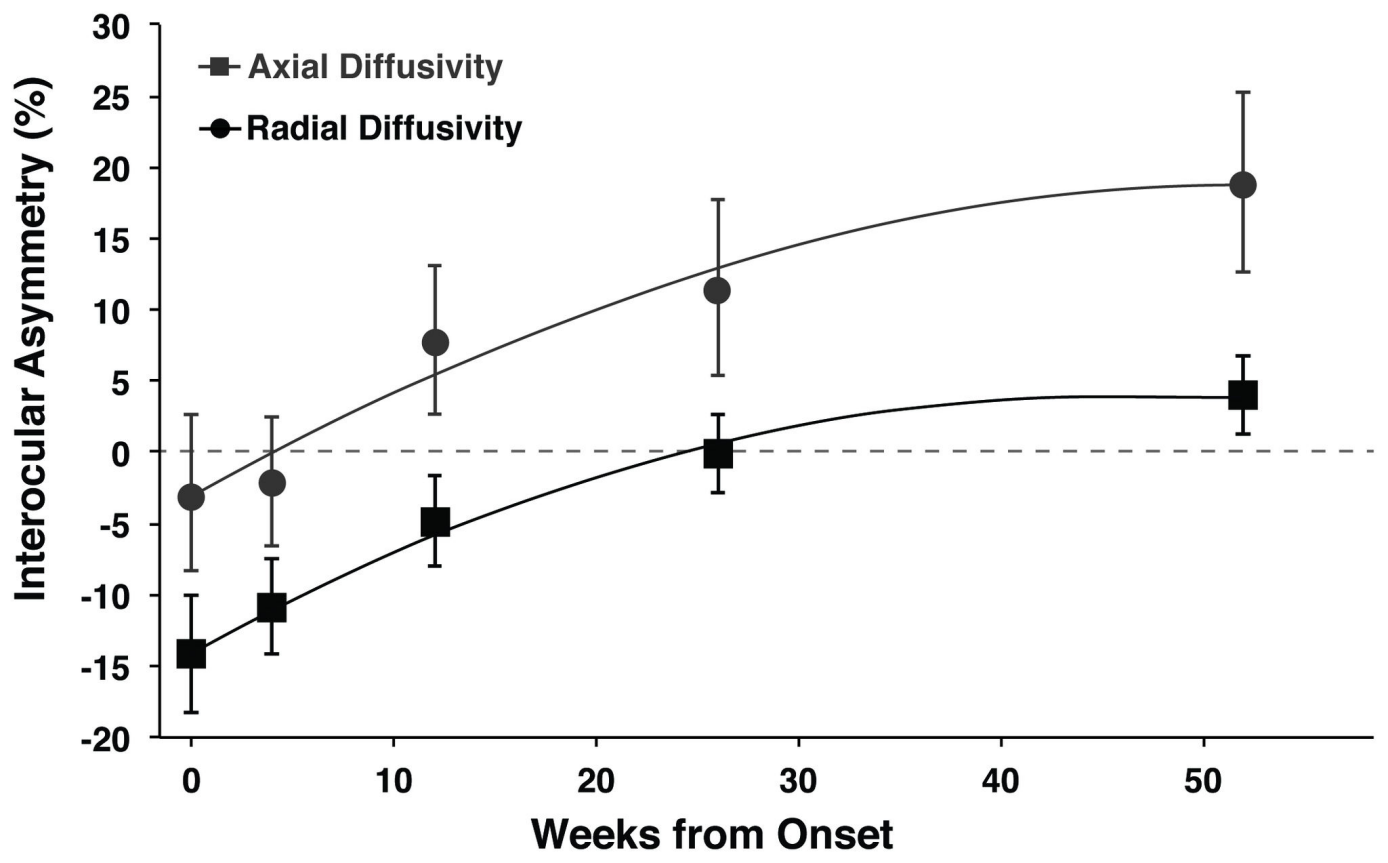
Timecourses for axial and radial diffusivity asymmetry over 12 months. Both timecourses were fit with quadratic lines of best fit (AD: *y* = -0.007*x* ^2^ + 0.81*x* - 3.3; RD: *y* = -0.008*x* ^2^ + 0.77*x* - 13.8). Error bars indicate 95% confidence intervals for the mean. * Significant interhemispheric asymmetry in axial diffusivity (p<0.05). # Significant interhemispheric asymmetry in radial diffusivity (p<0.05). UE = Unaffected Eye.

Mixed-effects two-way ANOVA revealed a significant time-point and optic nerve clinical status interaction (Wilk’s Lambda = 0.58, F_4,63_ = 11.56, *p* < 0.005). AD in affected nerves was reduced compared to unaffected nerves at baseline (-26%, 95% CI -34 to -18), one (-20%, -27 to -11) and three months (-9%, -15 to -3), was not different from unaffected at six months, and was increased compared to unaffected at 12 months (6%, 1 to 11). RD was increased compared to unaffected nerves at three (6%, 0.2 to 11), six (9%, 3 to 14) and 12 (16%, 10 to 21) months but did not change acutely and therefore was not further investigated in this analysis due to our specific interest in early markers of axonal and clinical outcomes. 

### Early optic nerve DTI changes and concurrent visual dysfunction (Table 3)

**Table 3:** Correlation analyses comparing early axial diffusivity with concurrently measured markers of inflammation and visual function.

	Axial Diffusivity	RNFLT*	mfVEP amplitude*	HCLA#	LCLA#
	R / ρ	0.22	**0.46**	**0.44**	0.00
Baseline	*p*	0.19	**0.007**	**0.006**	0.99
	N	37	**34**	**37**	37
	R / ρ	0.20	0.29	0.12	0.21
1 month	*p*	0.23	0.10	0.49	0.22
	n	36	33	36	36
	R / ρ	-0.18	0.06	0.04	-0.01
3 months	*p*	0.28	0.74	0.81	0.96
	n	37	32	36	37

^*^ Pearson parametric correlation

# Spearman rank correlation

RNFLT = retinal nerve fibre layer thickness
mfVEP = multi-focal visual evoked potential
HCLA = high contrast letter acuity
LCLA = low contrast letter acuity

Reduction in AD at baseline was associated with acute loss of visual function measured using mfVEP amplitude (R = 0.46, p = 0.007), HCLA (ρ = 0.44, p = 0.006) and Ishihara colour vision (ρ = 0.31, p = 0.05). 

### Early optic nerve DTI changes and axonal outcomes (Table 4)

**Table 4:** Correlation analyses comparing early axial diffusivity with axonal outcomes at six and 12 months.

Axial Diffusivity		6 months		12 months	
		RNFL	mfVEP amplitude	RNFL	mfVEP amplitude
	R	0.07	0.14	0.15	0.21
Baseline	*p*	0.70	0.45	0.39	0.22
	n	35	32	37	35
	R	**0.38**	**0.41**	**0.44**	**0.48**
1 month	*p*	**0.037**	**0.019**	**0.008**	**0.003**
	n	**34**	**32**	**36**	**36**
	R	0.29	0.19	0.20	0.07
3 months	*p*	0.34	0.29	0.24	0.69
	n	37	36	36	36

RNFL = retinal nerve fibre layer thickness mfVEP = multi-focal visual evoked potential

Despite the most pronounced change in AD being at baseline, the degree of AD reduction at baseline did not correlate with any axonal or clinical outcome measures at six or twelve months. However, at one month, a strong trend between reduced AD in affected nerves and RNFL thinning and mfVEP amplitude attenuation in affected eyes at six months (RNFL: R = 0.38, *p* = 0.04; mfVEP: R = 0.41, *p* = 0.02) was observed. Reduced 1-month AD correlated strongly with axonal outcomes at 12 months (RNFL: R = 0.44, *p* = 0.008; mfVEP: R = 0.48, *p* = 0.003). Regression equations for these significant correlations report that a 1% reduction in 1-month AD predicted a RNFL decrease of 13.8 % (95% CI -18.9 to -8.7) at 6 months and 13.6 % (-18.2 to -9.0) at 12 months, and a mfVEP amplitude decrease of 15.0 % (-24.6 to -5.5) at 6 months and 14.6 % (-22.5 to -6.8) at 12 months. 

### Early optic nerve DTI changes and clinical outcomes (Table 5)

**Table 5:** Correlation analyses comparing early axial diffusivity with clinical visual outcomes at six and 12 months.

Axial Diffusivity		6 months			12 months		
		HCLA	LCLA	Colour vision	HCLA	LCLA	Colour vision
	ρ	-0.12	-0.17	0.17	-0.14	-0.10	0.07
Baseline	*p*	0.49	0.30	0.32	0.42	0.56	0.69
	n	35	35	35	37	37	37
	ρ	-0.07	0.18	0.11	-0.17	0.07	-0.03
1 month	*p*	0.68	0.31	0.52	0.31	0.69	0.88
	n	34	34	34	36	36	36
	ρ	**-0.52**	0.23	**0.21**	**-0.41**	0.07	0.21
3 months	*p*	**0.001**	0.19	**0.24**	**0.011**	0.69	0.20
	n	**35**	35	35	37	37	37

HCLA = high contrast letter acuityLCLA = low contrast letter acuity

AD decreases at baseline and one month were not associated with any clinical visual outcomes at either six or 12 months. However, 3-month AD correlated with persistent loss of HCLA at 6 (ρ = -0.52, *p* = 0.001) and 12 months (ρ = -0.41, *p* = 0.01).  There was no correlation between 3-month AD and LCLA or colour vision at 6 and 12 months. 

### Optic Nerve AD normalisation and axonal and clinical outcomes (Figure 2)

**Figure 2:**
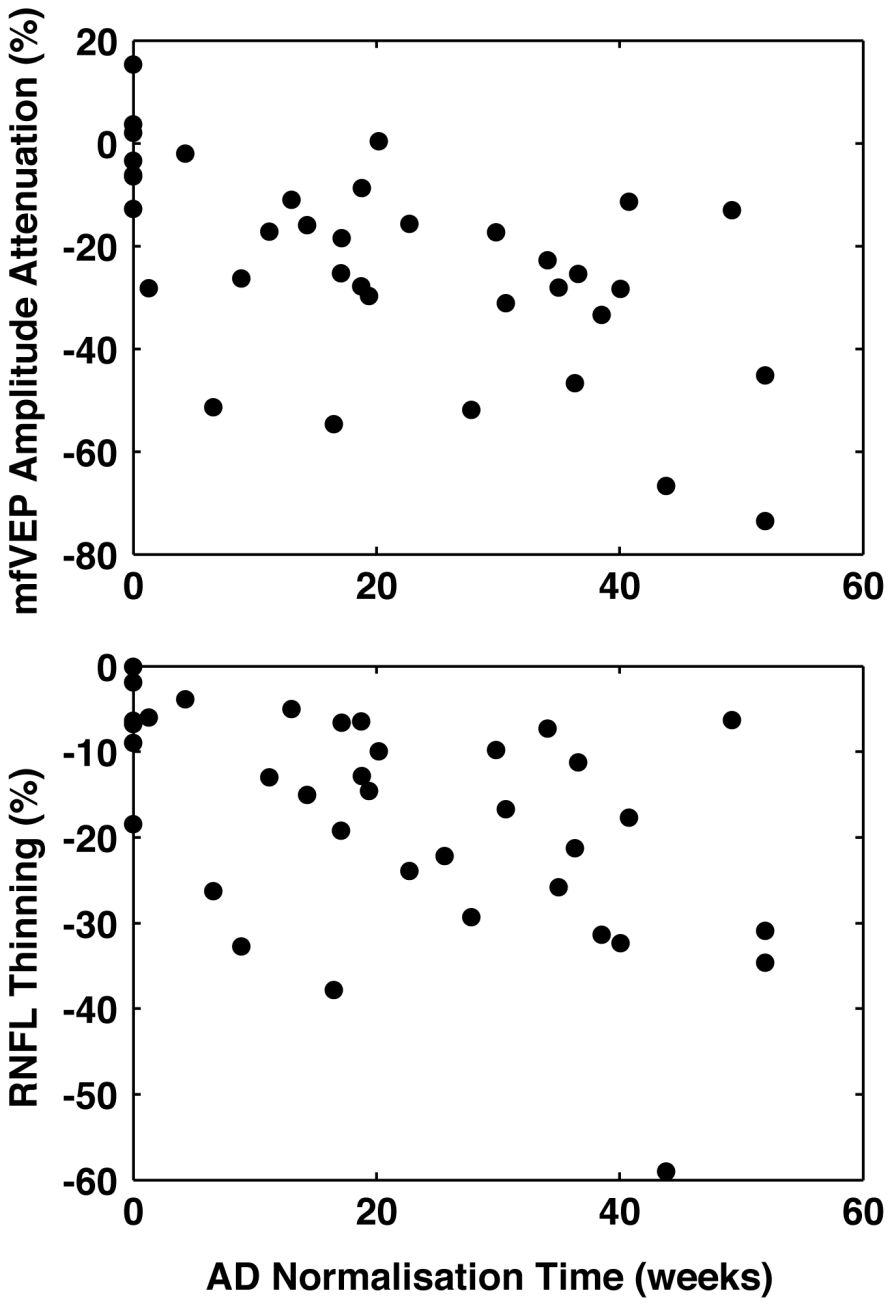
Optic Nerve AD normalisation and axonal outcomes. Prolonged AD normalisation time was significantly correlated with mfVEP amplitude loss (ρ = -0.59, p = 0.0002) and RNFL thinning (r = -0.54, p = 0.0005) at 12 months.

Quadratic fits to individual patient AD asymmetry time courses revealed a median time to normalisation of 18.9 weeks. Normalisation time was significantly correlated with mfVEP amplitude loss (ρ = -0.59, p = 0.0002) and RNFL thinning (r = -0.54, p = 0.0005) but not HCLA or LCLA at 12 months.

## Discussion

This study characterised changes in optic nerve DTI parameters in the first 12 months after ON in 37 patients either at high risk of, or with early MS. The selected cohort was clinically active and therefore relevant when interrogating surrogate outcome markers in the context of central demyelination. We reported that decreased AD at one month predicted axonal degeneration (RNFL thinning and mfVEP amplitude attenuation) at 12 months after acute ON with a strong trend to predict these outcomes at 6 months observed. Persistently decreased AD at three months predicted visual disability (loss of HCLA) at six and 12 months. Furthermore, we investigated the relevance of persistent AD reduction on axonal and clinical outcomes and found that prolonged time to normalisation significantly correlated with axonal degeneration at 12 months. 

Reduced AD associated with acute axonal injury is a relatively consistent finding in animal models [13,14], including mice with acute ON associated with experimental autoimmune encephalomyelitis (EAE) [24]. However, only one previous study by Naismith et al. [11] has reported reduced AD in human ON. That study reported acute reduction of AD, concordant with our results. However, the authors reported that the magnitude of baseline AD reduction was associated with axonal loss (VEP amplitude loss and RNFL thinning) and visual dysfunction (HCLA and contrast sensitivity) at six month follow-up. In contrast, we found that baseline AD reduction was strongly associated with concomitant loss of vision, most likely associated with the degree of acute inflammation. Furthermore, correlations between AD reduction and axonal outcomes were not evident until the one-month time-point in our study. Again in contrast to Naismith et al. [11] we found no association between early AD reduction and prolongation of mfVEP latency (a marker of demyelination [25] at 6 or 12 months. It should be noted that we applied mfVEP technology that is a substantial methodological improvement over the full-field VEP applied in other studies [11,26]. The mfVEP summates multiple responses from the peripheral visual field making it less susceptible to measurement of artefactually prolonged latencies [27]. We therefore conclude that early AD reduction is a marker relatively specific to axonal processes independent of the degree of ensuing demyelination. 

Several key differences in study design between this study and Naismith et al. [11] could in part account for the differences in observed results. Firstly, baseline measurements in the previous study were made between 1 - 31 days (median 12.5 days) after acute relapse compared to 1-14 days (median 5 days) in this study. Also, all subjects in this study were imaged within 48 hours of presentation and, in patients treated with IV methylprednisolone (76%), treatment was delayed until after baseline imaging. Therefore, baseline AD measurements in this study were probably confounded by acute inflammatory processes such as vasogenic oedema and cellular infiltration. While around half of the patients in the Naismith study received acute steroids, the timing of this in relation to initial scanning is not stated, making it difficult to appreciate the degree to which acute inflammation contributed to the baseline AD reduction in that cohort. Finally, the reported range of impairment (6/6 to no light perception) in the previous study suggested more severe optic nerve pathology in their cohort: this differential is consistent with the known increased baseline severity that accompanies ischemic [28] and simultaneous bilateral optic neuropathies [29] which comprised 6 out of 25 patients in their cohort. Patients in the current study were recruited according to more strict inclusion criteria permitting only inflammatory ON and excluding bilateral or recurrent cases. Therefore we consider our cohort to be highly representative of clinically isolated, non-ischemic unilateral ON, the most likely group to be recruited in trials of neuroprotective and remyelinating therapies, the second of which is currently in process (anti LINGO [30,31]; previously trialled erythropoietin [32]).

Significant associations between reduced AD at 3 months and HCLA were observed at six and 12 months. Subtle persistent visual symptoms are common after ON [33] but visual recovery is generally regarded as good [34] despite evidence of irreversible loss of myelin [7] and axons [22,35]. Naismith et al. [11] described a moderate correlation between 1-month AD and visual outcomes at 6 months using different visual outcome measures and visual recovery definitions to those used here. We used inter-ocular asymmetry of 2.5% LCLA scores because it has been shown by others to be the most sensitive clinical measure of visual loss [18,36], and is now commonly included in MS clinical trials [37]. Although mean visual impairment at baseline in our study (6/24) was similar to that described by Naismith et al. [11] (6/30), previously discussed differences in the types of optic neuropathies included in the study cohorts could also explain the somewhat discordant results. Notably, recent work suggests that compensatory neuroplasticity also contributes to visual recovery, independent of RNFL or VEP abnormalities, indicating the need for assessment of measures which interrogate different aspects of recovery and repair [38]. Overall, these results highlight the limitations of various methods of clinical visual testing and emphasize the need for defining outcome measures, particularly in clinical trial settings, using paraclinical endpoints such as RNFL thickness, now widely viewed as a sensitive and reliable direct measure of axonal loss [4].A novel finding of this study is that delayed normalisation of AD was associated with greater axonal injury at 12 months. By modelling individual patient AD time-courses using quadratic lines of best fit, we were able to estimate the time for AD in the affected optic nerve to return to equivalence with the unaffected nerve. We have previously shown that individual time-course modelling of optic nerve MTR could reveal sub-groups of patients based on putative optic nerve remyelination or degeneration and that these subgroups predicted axonal outcome [39]. The association between delayed normalisation and axonal outcome observed in this study has two important implications. Firstly, processes relevant to optic nerve axonal injury are potentially ongoing for weeks to months following acute relapse and visual recovery. As such, the window of opportunity for neuroprotective therapeutic intervention could be longer than previously suspected [3]. Secondly, the rapidity of AD normalisation could be a surrogate marker for the efficacy of such neuroprotective therapies. 

In conclusion, this work provides insights into the dynamic changes that occur in the optic nerve during and following acute ON in patients at high risk of developing MS. These analyses are of particular importance given the need for prognostic markers that can predict outcome early after disease onset. Reduced optic nerve AD could potentially meet this requirement both as a selection criterion at 1-month, and, by measuring normalisation time, as an early efficacy marker in trials of potential neuroprotective therapies. 

## References

[1] Barkhof F (1999) MRI in multiple sclerosis: correlation with expanded disability status scale (EDSS). Mult Scler 5: 283–286. doi:10.1177/135245859900500415. PubMed: 10467389. 10467389

[2] Rocca MA, Hickman SJ, Bö L, Agosta F, Miller DH et al. (2005) Imaging the optic nerve in multiple sclerosis. Mult Scler 11: 537–541. doi:10.1191/1352458505ms1213oa. PubMed: 16193891. 16193891

[3] Van der Walt A, Butzkueven H, Kolbe S, Marriott M, Alexandrou E et al. (2010) Neuroprotection in multiple sclerosis: a therapeutic challenge for the next decade. Pharmacol Ther 126: 82–93. doi:10.1016/j.pharmthera.2010.01.006. PubMed: 20122960. 20122960

[4] Frohman EM, Costello F, Stüve O, Calabresi P, Miller DH et al. (2008) Modeling axonal degeneration within the anterior visual system: implications for demonstrating neuroprotection in multiple sclerosis. Arch Neurol 65: 26–35. doi:10.1001/archneurol.2007.10. PubMed: 18195137. 18195137

[5] Klistorner A, Graham S, Fraser C, Garrick R, Nguyen T et al. (2007) Electrophysiological evidence for heterogeneity of lesions in optic neuritis. Invest Ophthalmol Vis Sci 48: 4549–4556. Available: http://eutils.ncbi.nlm.nih.gov/entrez/eutils/elink.fcgi?dbfrom=pubmed&id=17898277&retmode=ref&cmd=prlinks. doi:10.1167/iovs.07-0381. PubMed: 17898277. 17898277

[6] Fraser CL, Klistorner A, Graham SL, Garrick R, Billson FA et al. (2006) Multifocal visual evoked potential analysis of inflammatory or demyelinating optic neuritis. Ophthalmology 113: 323–323. PubMed: 16406544. 16406544 10.1016/j.ophtha.2005.10.017

[7] Klistorner A, Arvind H, Nguyen T, Garrick R, Paine M et al. (2008) Axonal loss and myelin in early ON loss in postacute optic neuritis. Ann Neurol 64: 325–331. doi:10.1002/ana.21474. PubMed: 18825673. 18825673

[8] Costello F, Hodge W, Pan YI, Eggenberger E, Coupland S et al. (2008) Tracking retinal nerve fiber layer loss after optic neuritis: a prospective study using optical coherence tomography. Mult Scler 14: 893–905. doi:10.1177/1352458508091367. PubMed: 18573837. 18573837

[9] Costello F, Hodge W, Pan YI, Freedman M, DeMeulemeester C (2009) Differences in retinal nerve fiber layer atrophy between multiple sclerosis subtypes. J Neurol Sci 281: 74–79. doi:10.1016/j.jns.2009.02.354. PubMed: 19303605. 19303605

[10] Henderson APD, Trip SA, Schlottmann PG, Altmann DR, Garway-Heath DF et al. (2008) An investigation of the retinal nerve fibre layer in progressive multiple sclerosis using optical coherence tomography. Brain 131: 277–287. doi:10.1093/brain/awm285. PubMed: 18056739. 18056739

[11] Naismith RT, Xu J, Tutlam NT, Lancia S, Trinkaus K et al. (2012) Diffusion Tensor Imaging in Acute Optic Neuropathies: Predictor of Clinical Outcomes. Arch Neurol 69: 65–71. doi:10.1001/archneurol.2011.243. PubMed: 21911658. 21911658 PMC3489058

[12] Naismith RT, Xu J, Tutlam NT, Snyder A, Benzinger T et al. (2009) Disability in optic neuritis correlates with diffusion tensor-derived directional diffusivities. Neurology 72: 589–594. Available: doi:10.1212/01.wnl.0000335766.22758.cd. Available online at: doi:10.1212/01.wnl.0000335766.22758.cd. PubMed: 19073948. 19073948 10.1212/01.wnl.0000335766.22758.cd PMC2672917

[13] Song S-K, Sun S-W, Ju W-K, Lin S-J, Cross AH et al. (2003) Diffusion tensor imaging detects and differentiates axon and myelin degeneration in mouse optic nerve after retinal ischemia. NeuroImage 20: 1714–1722. doi:10.1016/j.neuroimage.2003.07.005. PubMed: 14642481. 14642481

[14] Mac Donald CL, Dikranian K, Song S-K, Bayly PV, Holtzman DM et al. (2007) Detection of traumatic axonal injury with diffusion tensor imaging in a mouse model of traumatic brain injury. Exp Neurol 205: 116–131. doi:10.1016/j.expneurol.2007.01.035. PubMed: 17368446. 17368446 PMC1995439

[15] O'riordan J, Thompson A, Kingsley D (1998) The prognostic value of brain MRI in clinically isolated syndromes of the CNS. A 10-year follow-up. Brain. 10.1093/brain/121.3.495 9549525

[16] Polman CH, Reingold SC, Banwell B, Clanet M, Cohen JA et al. (2011) Diagnostic criteria for multiple sclerosis: 2010 revisions to the McDonald criteria. Ann Neurol 69: 292–302. doi:10.1002/ana.22366. PubMed: 21387374. 21387374 PMC3084507

[17] Group TONS (1991) The clinical profile of optic neuritis. Arch Ophthalmol 109 (1673–1678)

[18] Balcer LJ, Baier ML, Cohen JA, Kooijmans MF, Sandrock AW et al. (2003) Contrast letter acuity as a visual component for the Multiple Sclerosis Functional Composite. Neurology 61: 1367–1373. doi:10.1212/01.WNL.0000094315.19931.90. PubMed: 14638957. 14638957

[19] Baier ML, Cutter GR, Rudick RA, Miller D, Cohen JA et al. (2005) Low-contrast letter acuity testing captures visual dysfunction in patients with multiple sclerosis. Neurology 64: 992–995. doi:10.1212/01.WNL.0000154521.40686.63. PubMed: 15781814. 15781814

[20] Ishihara S (1982) Ishihara's Tests for colour blindness. 1982nd ed. Tokyo: Kanehara & Co., Ltd.

[21] Kurtzke JF (1983) Rating neurologic impairment in multiple sclerosis: An expanded disability status scale (EDSS). Neurology 33: 1444–1444. doi:10.1212/WNL.33.11.1444. PubMed: 6685237. 6685237

[22] Kolbe S, Chapman C, Nguyen T, Bajraszewski C, Johnston L et al. (2009) Optic nerve diffusion changes and atrophy jointly predict visual dysfunction after optic neuritis. NeuroImage 45: 679–686. doi:10.1016/j.neuroimage.2008.12.047. PubMed: 19162205. 19162205

[23] Bland JM, Altman DG (1986) Statistical methods for assessing agreement between two methods of clinical measurement. Lancet 1: 307–310. PubMed: 2868172. 2868172

[24] Wu Q, Butzkueven H, Gresle M, Kirchhoff F (2007) MR diffusion changes correlate with ultra-structurally defined axonal degeneration in murine optic nerve. NeuroImage 37: 1138–1147. doi:10.1016/j.neuroimage.2007.06.029. PubMed: 17689104. 17689104

[25] Brusa A, Jones SJ, Plant GT (2001) Long-term remyelination after optic neuritis: A 2-year visual evoked potential and psychophysical serial study. Brain 124: 468–479. doi:10.1093/brain/124.3.468. PubMed: 11222447. 11222447

[26] Hickman SJ, Wheeler-Kingshott CAM, Jones SJ, Miszkiel KA, Barker GJ et al. (2005) Optic nerve diffusion measurement from diffusion-weighted imaging in optic neuritis. AJNR Am J Neuroradiol 26: 951–956. PubMed: 15814951. 15814951 PMC7977095

[27] Halliday AM, McDonald WI, Mushin J (1972) Delayed visual evoked response in optic neuritis. Lancet 1: 982–985. PubMed: 4112367. 4112367 10.1016/s0140-6736(72)91155-5

[28] Hayreh SS, Zimmerman MB (2008) Nonarteritic anterior ischemic optic neuropathy: natural history of visual outcome. Ophthalmology 115: 298–305.e2 doi:10.1016/j.ophtha.2007.05.027. PMC2782939 17698200

[29] La Cruz De J (2006) Clinical profile of simultaneous bilateral optic neuritis in adults. Br J Ophthalmol 90: 551–554. doi:10.1136/bjo.2005.085399. PubMed: 16622084. Available online at: 10.1136/bjo.2005.085399 Available online at: PubMed: 16622084 16622084 PMC1857074

[30] Fu Q-L, Hu B, Wu W, Pepinsky RB, Mi S et al. (2008) Blocking LINGO-1 function promotes retinal ganglion cell survival following ocular hypertension and optic nerve transection. Invest Ophthalmol Vis Sci 49: 975–985. doi:10.1167/iovs.07-1199. PubMed: 18326721. 18326721

[31] Mi S, Miller RH, Lee X, Scott ML, Shulag-Morskaya S et al. (2005) LINGO-1 negatively regulates myelination by oligodendrocytes. Nat Neurosci 8: 745–751. doi:10.1038/nn1460. PubMed: 15895088. 15895088

[32] Sühs K-W, Hein K, Sättler MB, Görlitz A, Ciupka C et al. (2012) A randomized, double-blind, phase 2 study of erythropoietin in optic neuritis. Ann Neurol 72: 199–210. doi:10.1002/ana.23573. PubMed: 22926853. 22926853

[33] Cleary PA, Beck R, Bourque L, Backlund J, Miskala P (1997) Visual symptoms after optic neuritis. Results from the Optic Neuritis Treatment. Trial - Journal of Neuro-Ophthalmology: the Official Journal of the North American Neuro-Ophthalmology Society 17: 18–23. 9093956 10.1016/s0002-9394(14)70814-1

[34] Beck RW, Gal RL, Bhatti MT, Brodsky MC, Buckley EG et al. (2004) Visual function more than 10 years after optic neuritis: experience of the optic neuritis treatment trial. Am J Ophthalmol 137: 77–83. doi:10.1016/S0002-9394(03)00862-6. PubMed: 14700647. 14700647

[35] Kolbe SC, Marriott M, van der Walt A, Fielding J, Klistorner A et al. (2012) Diffusion Tensor Imaging Correlates of Visual Impairment in Multiple Sclerosis and Chronic Optic Neuritis. Invest Ophthalmol Vis Sci 53: 825–832. doi:10.1167/iovs.11-8864. PubMed: 22247457. 22247457

[36] Talman LS, Bisker ER, Sackel DJ, Long DA, Galetta KM et al. (2010) Longitudinal study of vision and retinal nerve fiber layer thickness in multiple sclerosis. Ann Neurol 67: 749–760. doi:10.1002/ana.22005. PubMed: 20517936. 20517936 PMC2901775

[37] Balcer LJ, Galetta SL, Calabresi PA, Confavreux C, Giovannoni G et al. (2007) Natalizumab reduces visual loss in patients with relapsing multiple sclerosis. Neurology 68: 1299–1304. doi:10.1212/01.wnl.0000259521.14704.a8. PubMed: 17438220. 17438220

[38] Jenkins TM, Toosy AT, Ciccarelli O, Miszkiel KA, Wheeler-Kingshott CA et al. (2010) Neuroplasticity predicts outcome of optic neuritis independent of tissue damage. Ann Neurol 67: 99–113. doi:10.1002/ana.21823. PubMed: 20186956. 20186956

[39] Wang Y, Van der Walt A, Paine M, Klistorner A, Butzkueven H et al. (2012) Optic nerve magnetisation transfer ratio after acute optic neuritis predicts axonal and visual outcomes. PLOS ONE 7: e52291. doi:10.1371/journal.pone.0052291. PubMed: 23272235. 23272235 PMC3525585

